# Knowledge of peri-menarcheal changes and a comparative analysis of the age at menarche among young adolescent school girls in urban and rural Cameroon

**DOI:** 10.1186/s12889-020-09787-y

**Published:** 2020-11-04

**Authors:** Atem Bethel Ajong, Nkengazem Nerry Tankala, Martin Ndinakie Yakum, Ikei Solange Azenoi, Bruno Kenfack

**Affiliations:** 1 Surgical, Obstetrics, and Gynaecology unit, Kekem District Hospital, Kekem, West Region Cameroon; 2 Department of Biochemistry, Faculty of Science, University of Dschang, Dschang, Cameroon; 3 Obstetrics and Gynaecology unit, Baham District Hospital, Baham, West Region Cameroon; 4 Medecins Sans Frontieres-Spain (MSF-OCBA), Epidemiology and Public Health, Old International Primary School Line NTA/Circular Road Junction Road by Dambua Road, Maiduguri, Nigeria; 5 Internal Medicine unit, Bafoussam Regional Hospital, Bafoussam, West Region Cameroon; 6 Dschang District Hospital, Dschang, West Region Cameroon; 7 Department of Obstetrics/ Gynaecology and Maternal Health, Faculty of Medicine and Pharmaceutical Sciences, University of Dschang, Dschang, West Region Cameroon

**Keywords:** Age at menarche, Knowledge, Peri-menacheal, Comparative analysis, Cameroon, Young adolescent girls

## Abstract

**Background:**

Menarche is an expected event that occurs during the development of every normal young girl. We designed this study to evaluate the knowledge of young school girls on puberty, menarche, and menstruation, and to update data on the age at menarche in a rural and urban setting in Cameroon.

**Methods:**

We conducted a cross-sectional survey from February to March 2017, targeting female students aged 9 to 16 years in Yaoundé (urban) and Bamougoum (rural). Participants were included using a randomised cluster sampling and data collected using an auto-administrable questionnaire. Student t-test or the Kruskal-Wallis test was used to compare means, and the odds ratio used as the measure of association between age at menarche and selected covariates.

**Results:**

1157 participants were included in the study; 49.3% from an urban setting and 50.7% from a rural setting. Regarding the knowledge of our participants on puberty, menarche, and menstruation, 67.20% of rural participants had good knowledge, whereas only 46.00% had good knowledge in the urban setting. Mean age at menarche was 12.76 ± 1.33 years, with the mean age at menarche in the urban setting of 12.48 ± 1.12 years and the rural setting of 13.03 ± 1.46 years. Mean age at menarche was significantly lower in participants aged below 14 years (*p*-value = 0.000), those with both parents alive (*p*-value = 0.0461), those whose fathers had skilled occupations (*p*-value = 0.005), those of urban resident (p-value = 0.000), and those who watched TV everyday (*p*-value = 0.030). Urban residence and age below 14 years were significantly associated with an earlier onset of menarche.

**Conclusion:**

Rural participants had better knowledge of puberty, menarche, and the menstrual cycle than their counterparts in the urban setting. The mean age at menarche over the last two decades has dropped by 7.4 and 4.2 months per decade in urban and rural Cameroon respectively. Mean age at menarche varies significantly with age group, urban/rural residence, state of both parents (both alive/at least one dead), occupation of the father, and frequency of watching TV. Age and urban/rural residence are associated with age at onset of menarche. The continually declining age at menarche is an alarm for future early menarche-linked morbidities.

**Supplementary Information:**

The online version contains supplementary material available at 10.1186/s12889-020-09787-y.

## Background

Menarche stands out amongst others as a crucial developmental stage in the lifespan of every normal young girl. It marks the onset of menstrual flow in the life of a developing young girl and comes with many fertility and reproductive implications. Though a commonly overlooked indicator in public health, menarche remains the key developmental marker of a girl’s transition from childhood into adulthood [1, 2]. It is described as an essential indicator of the physical, nutritional, and reproductive health status of every developing young girl [1, 3]. Puberty in young girls begins with thelarche (development of breast tissue), followed by pubarche (development of pubic hair), then menarche [4].

The mean age at menarche in Cameroon varies from urban to rural setting as was determined by Pasquet et al. using the “status quo” method in 1999 [3]. The study reported a mean age at menarche of 13.18 ± 1.08 years in the urban setting, 13.98 ± 1.55 years in the suburban setting, and 14.27 ± 1.65 years in the rural setting [3].

Over the last three decades, the age at menarche has experienced a consistent drop [2, 5]. Epidemiological evidence reveals multiple psychosocial and public health challenges that are due to the declining age of menarche. Early onset of menarche has been associated with early marriages [6, 7], premature parenthood, breast cancer [8, 9], development of myoma [10], psychosocial disorders [11, 12], metabolic syndrome (diabetes, hypertension, and obesity) [13–16], short stature [17], preeclampsia [18], poor academic performance and substance abuse [2, 19]. A substantial body of evidence from developed and developing nations agrees that early menarche (generally defined as menarche before the age of 12 years) increases the likelihood of adverse sexual and reproductive health outcomes including early pregnancy and childbearing, sexually transmitted diseases, early sexual initiation, and sexual violence among adolescent girls [1, 2, 20].

The age at menarche according to multiple epidemiological surveys has shown significant variations with economic status, educational attainment, race and ethnicity [21], place of residence (urban/rural) [3], nutritional status, and family sizes [2]. It is therefore a sensitive marker of many population indices including diet patterns, socio-economic status, geographical location, and environmental conditions.

Apart from the two studies by Pasquet et al. [3] and Monebenimp et al. [22] in 1999 on age at menarche, Studies on menarche and factors associated with menarche are still very few in Sub-Saharan Africa and particularly in Cameroon. The socio-economic and demographic changes over the years are likely to have been accompanied by changes in the age at onset of menarche. We therefore designed this study to update data on the current age at onset of menarche and to evaluate knowledge of young school girls on puberty, menarche and the menstrual cycle in a rural and urban setting in Cameroon, while doing a comparative analysis of the mean age at menarche across different groups.

## Methodology

### Study design

A school-based cross-sectional study was conducted in two regions of Cameroon from February to March 2017 targeting young adolescent female students aged 9–16 years. Data collected from these young girls (in Yaoundé and Bamougoum) were going to help us update information on menarche in urban and rural Cameroon. This was done using a pretested auto-administered questionnaire and the collected data analysed using Epi-Info version 7.2.2.16.

### Setting

The study was conducted in Yaoundé (urban) and Bamougoum (rural). Yaoundé is the administrative capital of Cameroon and is principally made up of the Mfoundi division (In the Centre Region of Cameroon). A cosmopolitan population characterises Yoaunde with about all the ethnic groups in Cameroon represented. Bamougoum on the other hand, is a rural area in Rural Bafoussam, Mifi division, West of Cameroon. The population is dominated by the Bamilekés (ethnic group) who are generally farmers and small traders with their closest urban centre being Bafoussam central town.

### Sampling

A multistep cluster sampling method was used in this study. Among the seven sub-divisions that make up the Mfoundi division, two were randomly selected for the survey (the names of the subdivisions were written on separate pieces of papers and folded, mixed and twoselected without replacement). All secondary schools in each of the two selected divisions were censored, their names written on separate pieces of papers, folded and mixed. One school was then selected randomly from each sub-division. The schools selected were Government Bilingual Practicing High School (GBPHS) Yaoundé and Government Bilingual High School (GBHS) Etoug-Ebe; Yaoundé. GBPHS and GBHS Yaoundé enrolled about 4800 and 6000 students respectively in the 2016/2017 academic year. As concerns the rural setting, due to the small number of schools, from the list of all secondary schools in Bamougoum, two were randomly selected. This was done by writing the names of all the schools on separate sheets of papers which were folded and mixed.. By this process, GBHS Bafoussam Rural and GBHS Nguache were selected. GBHS Bafoussam Rural and GBHS Nguache had an enrollment of 2500 and 3000 students respectively in the 2016/2017 academic year.

At the level of the selected schools, given that our study targeted female students aged 9–16 years (adapted age range for the status quo method of evaluating the mean menarcheal age) who had experienced menarche [2], based on information from the school registers, Form three to lower sixth classes were included in the study in both urban and rural settings. All female students within this class range who were below 9 years or above 16 years of age or who had not yet experience menarche were not included. In each form or level, if multiple classes existed, one class was randomly selected and included in the study. In a selected classroom, all eligible participants who accepted to participate were enrolled in the study.

### Procedure of implementation and data collection

When the protocol and data collection tools were ready, administrative authorisations were obtained from the selected schools and ethical clearance obtained from the institutional ethical review board of the University of Yaoundé I. The data collection tools were pretested in a sample of 15 students in a secondary school in Yaoundé (Faith Comprehensive Secondary School). A total of thirty (30) surveyors were recruited and trained on the consenting and data collection procedure in two training sessions of four hours each and then divided into six (6) teams each made up of five (5) members and headed by a team supervisor.

In each school, every selected class was assigned a team headed by a supervisor. Introduction and briefing on the topic of research was done by the supervisor of each team after which all eligible participants were given the information notice of the research to allow the students obtain written and signed consent of their parents/guardians back at home. All willing participants that had obtained the consent of their parents/guardians to participate were included in the survey after obtaining their verbal assent. The questionnaires were then submitted to each participant to fill with a clear notice of liberty to address questions to the research team members only. The participants were given as much time as they needed to complete the questionnaire, and all questions for clarification on the questionnaire answered. Data collected included; their Socio-demographic data, knowledge on puberty and menstruation, the onset of menstrual flow, and information on the age at menarche. During the submission of the filled questionnaire, the body weight (in kg) of each participant was taken using a digital weighing scale and registered in the questionnaire. During weight measurement, each participant removed her shoes, and any other dress she wore on her uniform (only the school uniform, which is very light in our setting, was used during weight measurement). The height (in meters) of each respondent was then taken in an erect position using a graduated height measuring device (see the questionnaire: Supporting information S1).

### Data management and analysis

All filled and submitted questionnaires were cross-checked for validity. Questionnaires that lacked vital information like the age of the participant and the age at menarche were excluded. Data were entered into a predesigned data entry sheet developed with the statistical software; Epi Info version 7.2.2.16. Proportions and their 95% confidence intervals were presented for categorical variables (occupation, level of education, religion … etc) while the mean or median was presented for continuous variables where applicable (age of the participant, age of menarche, and the number of household occupants … .etc). A participant was classified to have good knowledge if she had a 50% correct score on the questions evaluating the level of knowledge on puberty, menarche, and menstruation. The age at menarche was scategorised into two groups based on the median age at menarche registered in the study.

With a statistically significant threshold set at *p*-value = 0.05, and dependent on the result of the Bartlett’s test for inequality of population variances, different means were compared using either the T-test or the Mann-Whitney/Wilcoxon Two-Sample Test (Kruskal-Wallis test for two groups) where applicable. As concerns factors associated with menarche, the odds ratio and its 95% confidence interval were used as a measure of the strength of association between age at menarche (dichotomously scategorised based on the median age at menarche) and selected covariates. All variables with a *p*-value less than 0.5 from the bivariate analysis were all controlled one for the others in the multiple logistic regression model with a statistically significant threshold set at p-value = 0.05.

### Ethical considerations

Ethical approval for this study was obtained from the institutional ethical review board of the faculty of Medicine and Biomedical Sciences of the University of Yaoundé I. An information notice was given to each participant or their legal representatives. The written consent and verbal assent of the parent/guardian and participant respectively were obtained before data collection. Only willing and consenting participants were included in the survey. Confidentiality and the participant’s autonomy were respected.

## Results

### Socio-demographic characteristics of the participants

Out of the 1230 young girls contacted for the study, 53 denied participating (consent denied by their parents), giving a non-response rate of 4.31%. We excluded 20 questionnaires that were either wrongly filled or lacking vital information, and retained a total of 1157 participants (570 urban and 587 rural) for the study. The ages of the participants ranged from 10 to 16 years (No participant who was 9 years old was found to have started menstruation) with a median age of 15.0 years. The mean age of the participants was 15.1 years for rural dwellers and 14.8 years in urban dwellers. This age difference was statistically significant, with a *p*-value of 0.00 from unpaired t-test. Table 1 presents the socio-demographic characteristics of the participants. From Table 1, a majority of male parents of participants of the urban setting had attained higher education (52.98%), whereas, in the rural setting, a majority of male parents ended at primary school (33.73%). In the urban setting, most of the participants (54.21%) had household members ranging from 1 to 5 whereas, in the rural setting, most of the participants (37.31%) had household members above 10. In the urban setting, 75.30% of male parents/guardians had skilled occupations whereas, in the rural setting, only 47.00% had skilled occupations. Out of 1157 participants, 1118(96.62%) were Christians, and the rest were Muslims.

**Table 1:** Socio-demographic characteristics of the participants

Variable	modalities/options	URBAN, ***n*** = 570		RURAL, ***n*** = 587		Total, ***n*** = 1157	
		Frequency	Percentage	Frequency	Percentage	Frequency	Percentage
**Level of education** **of male parent/guardian**	never schooled	17	2.98	103	17.55	120	10.37
	Primary	77	13.51	198	33.73	275	23.77
	Secondary	174	30.50	151	25.72	325	28.09
	Higher	302	52.98	135	23.00	437	37.70
**Number of household** **Members**	1–5	309	54.21	151	25.72	460	39.76
	5–10	214	37.54	177	30.15	391	33.79
	10 and above	47	8.24	219	37.31	266	22.99
**Occupation of male** **Parents**	Skilled	429	75.30	276	47.00	705	60.93
	Unskilled	141	24.70	311	53.00	452	39.07

### Knowledge of participants on puberty, menarche, and menstruation

Regarding the knowledge of our participants as a whole, 67.20% of rural participants had good knowledge concerning puberty, menarche, and the menstrual cycle, whereas only 46.00% had good knowledge in the urban setting. There existed a very statistically significant difference in the level of knowledge in urban and rural settings (*p*-value< 0.001). The knowledge of the participants on puberty and menarche is presented in Table 2. From Tables 2, 91.40% and 92.80% of participants in urban and rural settings respectively had the correct definition of puberty. A Majority of participants (80.58% in the rural and 63.00% in the urban setting) had the corect knowledge concerning the age of onset of menarche (reporting that menarche occurs between 9 and 16 years).

**Table 2:** Knowledge of the participants on puberty and menarche

***Questions***	***responses***	***URBAN n = 570***		***RURAL n = 587***		***Total, n = 1157***	
		Frequency	Percent (%)	Frequency	Percent (%)	Frequency	Percent (%)
***What do you understand by Puberty?***	***Period of transition from childhood to adulthood***	521	91.40	545	92.80	1066	92.13
	***A period I can get pregnant***	39	6.80	35	6.00	74	6.40
	***A time that I am fit for marriage***	10	1.80	7	1.20	17	1.47
***At what age should a woman start menstruating?***	***Less than 9 years***	159	27.89	107	18.20	266	22.99
	***9- 16 years***	360	63.16	473	80.60	833	71.99
	***greater than 16 years***	51	8.94	7	1.20	58	5.01

Table 3 summarises the knowledge of the participants on the menstrual cycle. It was noticed that 78.40% of the rural participants knew menses flow for 2 to7 days as opposed to 86.70% in the urban setting. As concerns frequency of menstrual flow, 71.10% and 80.70% in the rural and urban setting respectively reported that menstrual flow occurs every 21 to 35 days***.***

**Table 3:** Knowledge of the participants on the menstrual cycle

Questions	Responses	RURAL n = 587		URBAN n = 570		Total, n = 1157	
		Frequency	Percent (%)	Frequency	Percent (%)	Frequency	Percent (%)
**For how many days should menses flow?**	**Less than 2 days**	114	20.00	72	12.30	186	16.08
	**2 to 7 days**	447	78.40	509	86.70	956	82.63
	**Greater than 7 days**	9	1.60	6	1.00	15	1.30
**How long is a menstrual cycle?**	**Less than 21 days**	147	25.80	106	18.10	253	21.87
	**21 to 35 days**	405	71.10	474	80.70	879	75.97
	**Greater than 35 days**	18	3.20	7	1.20	25	2.16

The knowledge of the participants on the premenstrual signs and symptoms is shown in Table 4. In the urban setting, 66.50% identified menstrual cramps, 13.30% nausea and vomiting, 23.30% increased breast size, 8.10% fever, 15.40% dizziness as possible signs and symptoms. In the rural setting, 72.10% identified menstrual cramps, 8.50% nausea and vomiting, 41.40% increased breast size, 10.60% fever, 11.40% dizziness.

**Table 4:** Knowledge of participants on the premenstrual signs and symptoms

***What are the possible signs/symptoms that can occur just before menstruation?***	***URBAN, n = 570***		***RURAL, n = 587***		***Total, n = 1157***	
	Frequency	Percent (%)	Frequency	Percent (%)	Frequency	Percent (%)
***Menstrual cramps***	379	66.50	423	72.10	802	69.32
***Nausea and vomiting***	76	13.30	50	8.50	126	10.89
***Increased breast size***	133	23.30	243	41.40	376	32.50
***Fever***	46	8.10	62	10.60	108	9.33
***Dizziness***	88	15.40	67	11.40	155	13.40

**Fig. 1:**
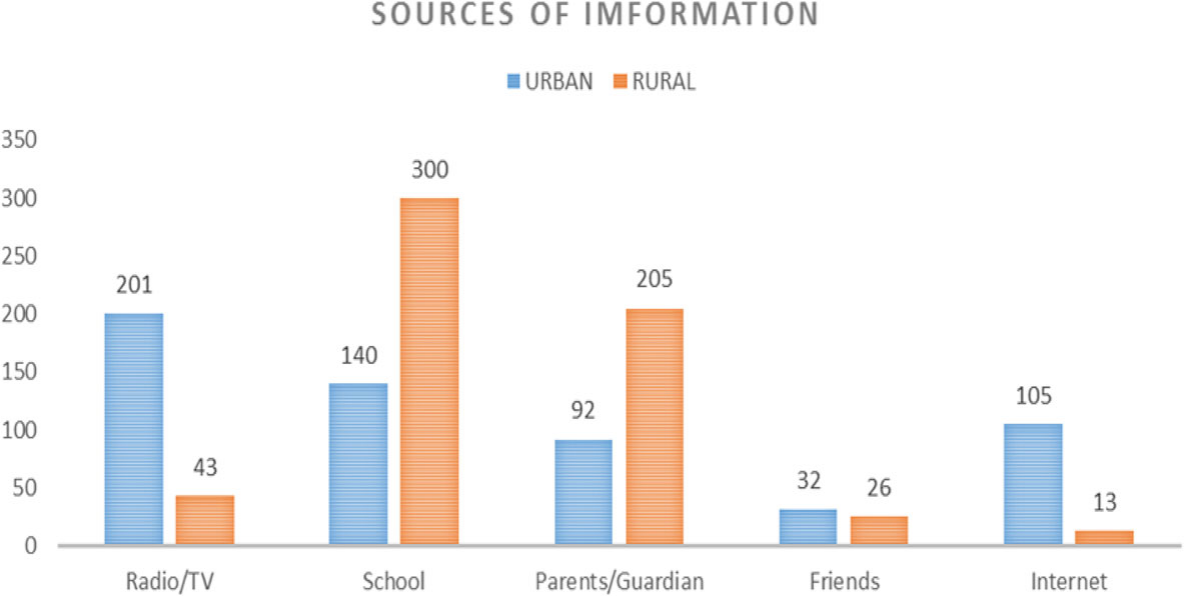
Sources of information of the participants on puberty, menarche and the menstrual cycle

Figure 1 summarises the sources of information of the participants on puberty, menarche, and the menstrual cycle (source of information on the horizontal axis and frequency on the vertical axis). Urban participants got information mostly from radio/TV 201(35.26%) whereas, in the rural setting, the primary source of information was from school 300(51.10%).

### Comparative analysis of age at menarche of the participant

The prevalence of early menarche (menarche before 12 years) was 14.87 [12.92–17.02] %. This prevalence was 16.32[13.51–19.57]% and 13.29[10.78–16.27]% in urban and rural setting, respectively. The mean age of the participants at menarche was 12.76 ± 1.33 years, with the mean age at menarche of 12.48 ± 1.12 years and 13.03 ± 1.46 years, respectively in the urban and rural settings. This mean age at menarche was significantly higher in the rural participants compared with participants in the urban setting (*p*-value< 0.001). The median and the modal age of the participants at menarche was 13 years (Cut-off considered for analysis of factors associated with menarche).

Table 5 presents a comparative analysis of the mean age at menarche in different groups. The mean age at menarche was significantly lower in participants with both parents alive compared to those with at least one parent dead (*p*-value = 0.0461). Participants with male parents who had skilled occupations attained menarche earlier compared to their counterparts with male parents having unskilled occupations (*p*-value = 0.005). Participants who watched television everyday attained menarche earlier compared to their counterparts who never watched television or did only on week-ends (p-value = 0.030). The mean age at menarche among participants with body mass index (BMI) greater than 25 kg/m^2^ was significantly lower than that among participants with BMI lower than 25 kg/m^2^ (*p*-value = 0.003). Participants in the urban setting attained menarche earlier than participants from the rural setting (*p*-value< 0.001). Also, girls below 14 years attained menarche earlier compared to their older counterparts (*p*-value< 0.001).

**Table 5:** Comparative analysis of the mean age at menarche stratified by some variables

V***ariables***	***Modality***	***Mean age (year)***	***SD (standard deviation)***	***p-value***
***State of parents***	Both alive	12.72	1.36	0.0461*^,μ^
	At least one dead	12.95	1.32	
***Age above 14 year***	Yes	13.02	1.39	0.000^£,μ^
	No	12.25	1.03	
***Occupation of male parents***	Skilled	12.67	1.27	0.005^£,μ^
	Unskilled	12.90	1.41	
***Male Parents’ level of education***	Not schooled and primary	12.89	1.34	0.100*
	Above primary	12.73	1.33	
***Watching of Television***	Never or only on week-ends	12.86	1.34	0.030*^,μ^
	Everyday	12.69	1.32	
***BMI***	Greater than 25 kg/m^2^	12.48	1.14	0.003^£,μ^
	Less than 25 kg/m^2^	12.94	1.46	
***Religion***	Christian	12.76	1.33	0.711*
	Muslim/others	12.83	1.41	
***Urban/Rural resident***	Urban	12.48	1.12	0.000^£,μ^
	Rural	13.03	1.46	

^£^ Mann-Whitney/Wilcoxon Two-Sample Test *p*-value, * t-Test *p*-value, ^μ^ Statistically significant *p*-value < 0.05

Table 6 presents factors associated with early onset of menarche among the participants both in simple and multiple logistic regression. On simple logistic regression, urban residence, age above 14 years, and BMI above 25 kg/m^2^ were significantly associated with an early age at menarche. Participants from the urban setting were 1.76 times more likely to haveattained menarche earlier compared with those from the rural setting (OR = 1.76[1.39–2.23], *p*-value = 0.000). In the same light, participants aged above 14 years were significantly less likely to have attained menarche earlier compared with those below 14 years (OR = 0.33[0.25–0.42], *p*-value = 0.000). Participants with a body mass index greater than 25 kg/m^2^ were significantly more likely to have an earlier menarche compared to those with a lower BMI (OR = 1.72[1.07–2.77], *p*-value = 0.024).

**Table 6:** Factors associated with age at menarche (Before 13 years)

Factors	Bivariate analysis			Multivariate analysis		
	OR	CI 95%	p-value	Adj OR	Adj CI 95%	*p*-value
Resident (urban/rural)	1.76	1.39–2.23	0.000*^,μ^	4.35	2.27–8.33	0,000^μ^
Level of education of male parent above primary (Y/N)	1.12	0.83–1.52	0.449*	0.10	0.49–2.01	0.997
Age above 14 years (Y/N)	0.33	0.25–0.42	0.000*^,μ^	0.30	0.17–1.52	0.000^μ^
Occupation of male parent (Unskilled/skilled)	0.80	0.62–1.01	0.066*	1.38	0.74–2.58	0.312
Christian (Y/N)	1.06	0.57–1.98	0.857			
Always eats to satisfaction (Y/N)	1.10	0.82–1.48	0.511			
Both parents alive (Y/N)	1.28	0.91–1.80	0.162*	0.48	0.22–1.05	0.066
Watches TV everyday	1.16	0.91–1.47	0.221*	1.49	0.87–2.53	0.143
BMI above 25Kg/m^2^	1.72	1.07–2.77	0.024*^,μ^	0.98	0.56–1.70	0.939

* Variables included in the multiple logistic regression model (*p*-value < 0.5), ^μ^Statistically significant *p*-value < 0.05

Upon control in a multiple logistic regression model, only age above 14 years and urban/rural residence remained statistically significant. The strength of association of the urban/rural setting with early menarche increased significantly. Participants from the urban setting were 4.35 times more likely to have attained menarche earlier compared with their counterparts in the rural setting (OR = 4.35[2.27–8.33], p-value = 0.000). In the same light, participants aged above 14 were 0.30 times less likely to have attained menarche earlier compared with their younger counterparts.

## Discussion

Menarche is a physiological event occurring during the development of every normal young girl. This study updates data on menarche in a rural and urban Cameroonian setting, goes further to evaluate the knowledge of young adolescent girls on puberty, menarche, and the menstrual cycle while identifying possible factors influencing the age of onset of menstrual flow in these girls.

Regarding the knowledge of our participants, 56.6% of participants had above 50% of the score (good knowledge) concerning puberty, age at menarche, and menstrual cycle. This finding is similar to that obtained in a Ghanaian study in 2016, which reported an average knowledge of 57% on menarche and the menstrual cycle [23]. The proportion of participants with good knowledge is unsurprisingly higher in this study, given that the target population was university students. Up to 67.20% of participants from rural settings had good knowledge, whereas only 46.00% had good knowledge in the urban setting. This difference may be because the primary source of information in the rural area was the school (51.10%) whereas in the urban participants it was the radio/TV and friends. The higher proportion of rural participants with good knowledge in our study questions the influence of the information source types on the knowledge of the participants. Probably, participants in the rural zone who have the school as their primary source of information were not influenced by diverse ideas in this field. Also, the quality of teaching care administered in the subject matter in both settings might be different. Our study however did not evaluate the quality of education administered to students in this domain, and therefore we cannot be conclusive in our reasoning.

Moreover, the mean age of participants was 15.1 years for rural dwellers and 14.8 years in urban dwellers. This age difference was statistically significant, with a *p*-value of 0.00 from unpaired t-test. Older girls have a higher propensity of being exposed to information on puberty and menstruation through parents’ education, studies in school, peer discussion, and other media. This might be a possible explanation of why rural-dwelling girls had better knowledge of puberty and menstruation than the urban-dwelling girls. Also, it is possible that our results might report a relatively lower level of knowledge given the nature of the designed questionnaire, the conditions under which the questionnaire was administered, and the age group considered.

The primary source of information on puberty, menarche, and menstrual cycle in the urban participants was the radio/TV (35.26%) whereas, in the rural area, the primary source of information was the school (51.10%). This is expected, given that the urban areas are generally associated with easy access to audiovisual signals. When we look at the proportion of students with good knowledge in rural and urban settings, we can state that the level of knowledge is still relatively low and even lower in the urban setting. Good knowledge has been associated with better attitudes and good attitudes with practices. This level of knowledge among already menstruating girls could be associated with poor practices as far as sexual behaviour and menstrual hygiene are concerned. Readapting or strengthening the course content of these young adolescent girls in their respective schools is indispensable in improving the quality of knowledge of these young girls. Parental home counselling and sensitisation of the young girl is also of utmost importance.

The mean age at menarche in our study was 12.76 ± 1.33 years, with a mean age at menarche in the urban setting (Yaoundé) of 12.48 ± 1.12 years and the rural setting (Bamougoum) of 13.03 ± 1.46 years. When compared, this difference was statistically significant, with a *p*-value of less than 0.001. A study carried out in Cameroon in 1999 by Pasquet et al. reported an average age at menarche of 13.18 ± 1.08 years in urban, 13.98 ± 1.55 years in the suburban area, and 14.27 ± 1.65 years in the rural area [3]. The trend is similar to the trend recorded in this study, given that the age at menarche decreased from rural, through suburban to the urban setting. Compared to the results of Pasquet et al. the mean age at menarche has dropped by 1.24 years and 0.7 years in the urban and rural settings respectively over the last two decades in Cameroon. This drop of the mean age at menarche corresponds to a decrease of 7.4 and 4.2 months every decade in the urban and rural setting respectively. We are however not very conclusive about this decrease as these surveys were carried out among participants with different age limits. This declining trend of the age at menarche has also been reported in population studies carried out in Gambia [24], China [25], and Vietnam [26]. All the above-cited literature presented significant differences between the ages of menarche in urban and rural settings.

This declining trend of the age at menarche is in line with the high prevalence of early menarche recorded in this survey. According to our findings, 14.87% of these young girls attained menarche before the age of 12 years. This progressively early onset of menarche has been described to be associated with the development of multiple gynaecological morbidities like the development of myoma [10], cardiovascular disease [15, 27], metabolic syndrome [14], type 2 diabetes [16], preeclampsia [18], and various forms of cancer [8, 9].

Comparative analysis of the mean age at menarche across different groups showed that the mean age at menarche was significantly influenced by the state of both parents (both alive/ at least one dead), the current age of the participants, the occupation of the parents, frequency of watching television, and body mass index of the participants. With both parents alive, the standard of living of the young girl is likely to be better given the increased likelihood of a better socio-economic status [2]. According to a study on age at menarche and socio-economic status in Poland, girls from families with high socio-economic status experienced menarche at an earlier age than girls from families with lower socio-economic status [28]. Also, multiple studies have agreed that higher BMI is associated with an increased likelihood of early menarche [29–31]. Watching television has been described to have a significant influence on the sexual development and behaviour of adolescent girls. It has also been associated with an increased likelihood of early menarche [32, 33]. Participants with parents having skilled occupations are generally more likely to experience an earlier menarche compared to those whose parents have unskilled occupations. This left us hypothesising that participants with parents having skilled occupations were more likely to have a better socio-economic state compared to others. This could be a plausible explanation for the difference. Studies have however presented contrasting findings; stating that the age at menarche did not depend on the parent’s occupation [2].

When we evaluated by logistic regression with age at menarche categorised (Menarche before 13 years), we found that only age and urban/rural residence were significantly associated with age at menarche. As already discussed above, urbanisation is by far the strongest factor associated with menarche among young girls. Girls in the urban setting were more than four times more likely to experience menarche earlier compared to their counterparts in rural settings even upon control for multiple variables. The growing urbanisation of the different rural zones in Cameroon is likely to be associated with a very significant drop in the age of first menstrual flow in Cameroon. This will mean higher exposure to early menarche-linked morbidities in the future. Older girls reported a significantly higher mean age at menarche compared to their younger counterparts. Logistic regression results confirmed age as a statistically significant factor associated with age at menarche. The impact of rapid urbanisation over the years and the rapid changes in the methods of child upbringing associated with an increasing prevalence of childhood obesity could explain this [34].

The level of education of the male parent and BMI were significant factors only on simple logistic regression. These factors have already been reported to be associated with menarche in multiple studies [2, 30, 31]. These two factors are possible confounders of the urban/rural residence, given that parents in the urban setting are likely to be more educated than their counterparts in the rural setting. Also, city dwellers end up with a significantly larger BMI compared to those in rural settings [35].

The data herein should be interpreted with care. Information bias was likely to affect our results given that we conducted the study on already menstruating females. The data given to us was therefore retrospective and could be subjected to errors (failure of memory). Moreover, the participants could lie given that some of the questions were personal and did explore their reproductive development. The data collected in this survey does not permit us to evaluate all possible factors associated with earlier onset of menarche. Other possible factors like level of education and occupation of the mother, the wealth index of the family, and nutritional status were not evaluated in this study. Also, the cross-sectional design of the study does not permit us to establish cause-effect relations between variables. The findings in this research are not representative of all urban and rural zones in Cameroon; more extensive studies representing all the rural and urban zones in Cameroon are necessary. In addition, the difference observed between the urban and rural population in this study could be bigger given that the population of city dwellers is generally cosmopolitan (case of Yaoundé) compared to the case of Bamougoum (dominated principally by the Bamilekés; the major ethnic group of the West Region of Cameroon).

## Conclusion

Even though young girls in Bamoungoum (rural setting) have better knowledge of puberty, menarche, and the menstrual cycle, the overall level of knowledge in both settings is relatively good. The mean age at menarche of menstruating girls aged 9–16 years is 12.76 ± 1.33 years, with a mean age at menarche of 12.48 ± 1.12 years and 13.03 ± 1.46 years respectively in the urban and rural settings. The mean age at menarche over the last two decades in Cameroon has dropped by 7.4 and 4.2 months in the urban and rural settings respectively. The mean age at menarche depends on some factors like urban/rural residence, state of the parents (both alive/ at least one dead), the occupation of the male parent, age group, frequency of watching TV, and body mass index. Upon control for confounders, urban residence and age below 14 years were significantly associated with earlier onset of menarche.

Parental home counselling and sensitisation, associated with focused reinforcement of their course content on puberty, menarche, and the menstrual cycle is indispensable in stepping up the knowledge of these young girls. Declining age at menarche serves as an essential indicator of the future burden of gynaecological, and cardio-metabolic, morbi-mortality.

## Supplementary Information

**Additional file 1: Supporting information file (S1).** Questionnaire on menarche.

**Additional file 2:** **Supporting information file (S2).** Database containing “status quo” data on the age of menarche in rural and urban Cameroon.

## Data Availability

The datasets used and/or analysed during the current study are available in the database submitted as supporting information (S2).

## Abbreviations

AOR: Adjusted Odds Ratio
BMI: Body Mass Index
CI: Confidence Interval
GBPHS: Government Bilingual Practicing High School
GBHS: Government Bilingual High School
OR: Odds Ratio
