# Supplementary material for: Knowledge of peri-menarcheal changes and a comparative analysis of the age at menarche among young adolescent school girls in urban and rural Cameroon
Source: BMC Public Health. 2020 Nov 4;20:1661. doi: 10.1186/s12889-020-09787-y (PMC7641860; doi:10.1186/s12889-020-09787-y)
Supplement: Supplementary file 1 — Additional file 1: Supporting information file (S1). Questionnaire on menarche. [file 12889_2020_9787_MOESM1_ESM.docx]

**Questionnaire for evaluation of the knowledge of perimenarcheal changes and age at menarche among female adolescents**

Date…………………………… Code of participant: …/…/…/…/…

**Socio-demographic data**

1. Age: …………………………………………………………………………………………
2. State of parents: both alive
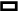
 one alive
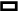
 both dead
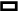

3. Parents/Guardians level of education (father): never schooled
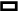
 primary
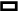
secondary
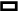
 higher
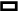

4. Number of household members………………
5. Occupation of parents (father): skilled
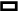
 unskilled
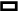

6. Religion: Christian Muslim Atheist Animist

**Knowledge**

1. What do you understand by puberty : period of transition from childhood to adulthood a period I can get pregnant a time that I am fit for marriage
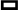

2. At what age should a woman start seeing her menses? Less than 9years
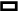
 9- 16years
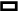
 greater than 16years
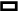

3. For how many days should menses flow? Less than 2days
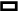
2-7days
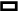
 more than 7days
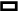

4. How long is a menstrual cycle (after how many days do the next menses start)? less than 21days
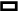
 21-35days
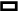
 more than 35days
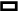

5. What are the premenstrual signs and symptoms that you know? Menstrual cramps
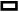
 Nausea and vomiting
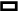
 increased size of breast
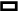
 diarrhoea
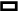
fever
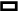
dizziness
6. What are the sources of your information? radio/tv
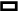
school
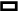
parents/guardian
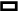
 friends
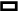


**Practical evaluation of factors affecting the age of menarche and clinical profile of participants**

1. When did you have your first menses………………………………………………(year )
2. How long is your menstrual cycle (after how many days do the next menses start)? less than 21days
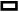
21-35days
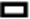
 more than 35days
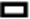

3. What are the premenstrual signs and symptoms that you usually have? menstrual cramps
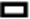
 Nausea and vomiting
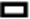
 increased size of breast
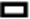
 diarrhoea
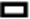
 fever
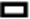
dizziness
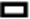

4. if menstrual cramp when did you start having it: the very period I started menstruating
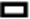
 within a year
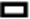
 more than a year after my first menses
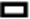

5. What activities do you usually perform out of school? Farm work
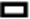
sport
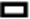
 watch TV
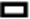
 sell with my mother
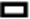
play games
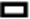
 others………………………………………………………………
6. How much time do you watch Television? Never
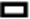
 only on weekends
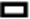
 Everyday
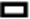

7. How much time do you read after school? Never
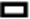
 everyday
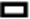
 only in school evaluation period
8. How often do you eat to your satisfaction? Never
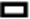
 at the beginning of the month
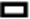
 two times a week
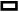
 often
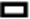
, others……………………………………………………………………
9. What type/class of food do you consume most? carbohydrate (cocoyam, rice, cassava etc)
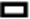
 protein( meat, fish etc)
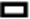
 vegetable
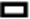
 lipids (fats)
10. Are you sexually active?
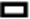
 Yes
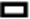
 No. If yes, is this before or after your first menses? ...............
11. Do you know the age in which your mother had her first menses?
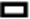
 yes
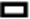
 no if yes when…………………………………….
12. Body weight……………………….height………………….BMI…………………………………
